# Distinct DC subsets are not equally susceptible to HTLV-1 infection

**DOI:** 10.1186/1742-4690-11-S1-O20

**Published:** 2014-01-07

**Authors:** Sandrine Alais, Audrey Glaize, Anne Cachat, Florian Lamouche, Renaud Mahieux, Hélène Dutartre

**Affiliations:** 1 Oncogenèse Rétrovirale, label Ligue Nationale Contre le Cancer, CIRI, Labex Ecofect, INSERM U1111-CNRS UMR5308, Ecole Normale Supérieure, Université Lyon 1 (UCLB) Lyon, Cedex 07, France

## 

Beside lymphocytes, HTLV-1 can infect monocytes, blood- or monocytes-derived dendritic cells (DCs) and plasmacytoïd DCs in vitro. Considering that DCs might be the first cells to be infected during primo infection, we hypothesize that they may constitute viral reservoirs and eventually spread the virus to surrounding lymphocytes. Interestingly, ATL and HAM/TSP diseases display opposite immunological features, i.e. an impaired CTL response versus a chronic inflammation respectively. Since DCs are major effectors of both innate and adaptative immune responses, infection of specific DC subsets and the subsequent alteration of their functions might also lead to the orientation of the adaptive immune response towards either viral tolerance associated with impaired CTL responses or chronic inflammation, and thus directly participate to the determination of the infection outcome. Using various cytokines cocktails, we therefore generated distinct monocytes-derived DC (MDDC) subsets and infected these cells with HTLV-1 biofilm. We first show that the different MDDC subsets are not equally susceptible to HTLV-1 infection, as measured by FACS analyses and real time PCR assays. We then show that following infection, DC activation or IFN alpha production are not affected by infection in the MDDC subtypes tested. However, while DC maturation alters their susceptibility to the virus, we demonstrate that IFN alpha treatment does not. Finally, the ability of MDDC subsets to transmit HTLV-1 to T-cells will be discussed. Taken together, our results suggest that differential susceptibility of various DC subsets to HTLV-1 infection could differently shape immune responses and therefore affect viral pathogenesis.

